# Evolution of CPITN Index in Relation to Chlorhexidine Mouthwash Use in Patients with Deflective Occlusal Contacts

**DOI:** 10.3390/bioengineering12111140

**Published:** 2025-10-22

**Authors:** Ximena Anca Nicolae, Elena Preoteasa, Cătălina Murariu Măgureanu, Ruxandra Moraru, Cristina Teodora Preoteasa

**Affiliations:** 1 Department of Dental Prosthodontics, Faculty of Dental Medicine, “Carol Davila” University of Medicine and Pharmacy, 041313 Bucharest, Romania or dr_elena_preoteasa@yahoo.com (E.P.); catalina.magureanu.murariu@umfcd.ro (C.M.M.); 2 Department of Dental Public Health and Primary Dental Care, Institute of Dentistry, Faculty of Medicine and Dentistry, “Queen Mary” University of London, London E1 2AD, UK; i.r.moraru@qmul.ac.uk; 3 Department of Scientific Research Methodology and Ergonomics, Faculty of Dental Medicine, “Carol Davila” University of Medicine and Pharmacy, 041313 Bucharest, Romania

**Keywords:** adjunctive periodontal therapy, chlorhexidine, CPITN, occlusal trauma, periodontal disease

## Abstract

Background/Objectives: Occlusal trauma does not initiate periodontitis but may accelerate its progression when inflammation is present. Chlorhexidine (CHX) mouthwash is widely used as an adjunct to periodontal therapy, although its effectiveness in patients with occlusal trauma is insufficiently documented. This study aimed to evaluate the effect of CHX mouthwash on periodontal status in patients with deflective occlusal contacts, in the absence of occlusal adjustments. Materials and Methods: This observational prospective study analyzed data from 52 patients (20 males, 32 females; mean age 41.35 years). Periodontal status was assessed using the Community Periodontal Index of Treatment Needs (CPITN) at baseline, 3 months, and 6 months. Patients were divided into groups based on CHX use and concentration. Statistical analysis evaluated intra- and intergroup variations. Results: Patients using CHX demonstrated statistically significant improvements in CPITN scores at 3 months for all sextants except sextant 5 (*p* < 0.05). Between 3 and 6 months, further significant improvement was observed only for sextant 6 and for the overall score. In contrast, patients without CHX showed a slight trend toward worsening CPITN values, with no statistically significant differences over the same period. Conclusions: CHX mouthwash significantly improved periodontal parameters in patients with occlusal trauma during the first three months of use. However, improvements plateaued after this period, highlighting the short-term benefits and limitations of CHX. These findings support the adjunctive role of CHX in managing periodontal disease associated with occlusal trauma but reinforce the necessity of mechanical occlusal correction for long-term stability. The CPITN index provides moderate clinical utility compared with full-mouth clinical periodontal measurements.

## 1. Introduction

Occlusal trauma refers to excessive or abnormal forces applied to the teeth and their supporting structures (periodontium) during chewing or other jaw movements involving occlusal contacts [1]. Periodontitis, by contrast, is an inflammatory disease that damages the soft tissues and the alveolar bone [1]. According to The Glossary of Prosthodontic Terms, Ninth Edition (GPT-9), occlusal trauma is an abnormal force applied to the teeth and to the periodontium, by functional or parafunctional forces, causing damage to the attachment system of the periodontium by exceeding its adaptive and reparative capacities; it may be self-limiting or progressive [1]. Occlusal trauma may exacerbate bone loss and tooth mobility, thereby accelerating the progression of periodontal disease [2].

While there is consensus that trauma from occlusion does not initiate chronic periodontitis, its role in the progression of periodontal disease remains a subject of debate. According to the American Academy of Periodontology, occlusal trauma may occur either independently of, or concurrently with, inflammatory periodontal conditions [3]. However, other authors affirm that occlusal trauma is strongly associated with periodontitis, and while not causing periodontitis, can significantly worsen the condition by accelerating bone loss and increasing tooth mobility in the presence of existing periodontal disease. [3] Occlusal forces lead to loss of the inorganic matrix of alveolar bone in the cervical region, compromising tissue integrity, potentially promoting the apical migration of inflammatory exudate related to biofilm, and facilitating direct invasion of the periodontal ligament [4].

Similar to periodontitis, occlusal trauma typically involves episodes of acute damage alternating with periods of adaptation or stabilization [3]. Periodontal tissue breakdown may result from either inflammatory or degenerative mechanisms [4]. Inflammatory destruction refers to the breakdown of periodontal tissues caused by the host immune response to bacterial biofilm [4]. It is characterized by loss of connective tissue attachment, apical migration of the junctional epithelium, and alveolar bone resorption associated with the formation of periodontal pockets [4]. This process is primarily microbiologically driven and mediated by inflammatory cytokines and osteoclastic activity [5]. Degenerative destruction, in contrast, results from excessive or abnormal occlusal forces acting on the teeth and their supporting structures [4]. It manifests as compression and hyalinization of the periodontal ligament, widening of the periodontal ligament space, and localized bone resorption without pocket formation [4]. This is a mechanical, non-infectious process reflecting the periodontium’s adaptive response to overload. When both processes overlap, they generate a ‘combined lesion’ of periodontitis and occlusal trauma, in which microbial inflammation and mechanical overload act synergistically to accelerate tissue breakdown [3,4].

However, the development of this combined lesion depends on the temporal alignment of the disease stages. If occlusal trauma is in an adaptive or remodeling phase while periodontitis is active, or vice versa, the combined lesion is unlikely to form [4]. This suggests that although biofilm-induced inflammation and occlusal trauma may coexist, they do not always result in compounded tissue destruction. Consequently, suprabony pockets and horizontal bone loss may occur alongside trauma without producing a true combined lesion [4,6,7].

Although occlusal trauma does not initiate periodontitis, it can exacerbate attachment loss in teeth with compromised periodontal support [8]. As such, occlusal interventions should be considered selectively, based on clear clinical indicators such as pathological tooth mobility, deflective occlusal contacts, or evidence of progressive tooth migration [8]. Therapeutic measures may include occlusal adjustment to eliminate interferences, as well as splinting in cases of functional impairment due to mobility [8]. These interventions should always be implemented alongside meticulous biofilm control and anti-inflammatory periodontal therapy [8].

Beyond its antimicrobial applications, the success of periodontal therapy also depends on mechanical and functional factors, such as occlusal load. Therefore, this study explores both microbial control using CHX and the influence of occlusal trauma on periodontal stability. Reducing tooth mobility may enhance the effect of periodontal therapy [9].

In dental practice, chlorhexidine (CHX) is employed both therapeutically and prophylactically [10,11]. An optimal dosage of 18–20 mg per application has been identified to provide maximum antimicrobial efficacy while minimizing adverse effects [12]. CHX exhibits rapid bactericidal and antifungal properties, maintaining its effectiveness even at low concentrations [11,12,13]. It is active against both aerobic and anaerobic bacteria and possesses virucidal activity against DNA and RNA viruses [12]. Specifically, CHX can inactivate lipid-enveloped viruses such as HIV, influenza A, parainfluenza, hepatitis B, herpes simplex, and cytomegalovirus [12].

Chlorhexidine (CHX) has long been regarded as the gold standard among antiseptic mouth rinses due to its broad-spectrum antimicrobial activity and strong oral substantivity [11,13]. Its mechanism of action involves membrane disruption, leading to leakage of intracellular contents and bacterial cell death. In clinical practice, CHX is employed across a variety of dental procedures, including periodontal therapy, surgical site disinfection, endodontic irrigation, and management of peri-implantitis [11,14]. CHX is available in various concentrations and delivery systems—from mouthwashes to gels and varnishes—each tailored for specific clinical applications. It is frequently used as an adjunct to non-surgical periodontal therapy, particularly in situations where mechanical plaque control is compromised, such as immediately following non-surgical or surgical periodontal therapy [11]. CHX has been shown to reduce plaque accumulation and gingival inflammation effectively when used short-term, typically being used for 2 to 4 weeks [13]. However, its long-term use is limited by well-documented adverse effects, including extrinsic tooth or tongue staining, mucosal irritation, enhanced calculus formation, and altered taste sensation [11,13,14,15].

This study aims to evaluate whether, in patients with deflective occlusal contacts, in the absence of any occlusal interventions, the use of chlorhexidine mouthwash can improve periodontal status. Although chlorhexidine has been extensively studied in periodontal therapy, its specific role in patients presenting occlusal trauma without concurrent occlusal correction remains insufficiently documented. The present study introduces an original perspective by analyzing the evolution of the CPITN index in this unique clinical context, thereby integrating microbial and functional factors within periodontal assessment.

## 2. Materials and Methods

This study was approved by the Research Ethics Committee of Carol Davila University of Medicine and Pharmacy (protocol code PO-35-F-03 and date of approval: 14 March 2025).

This study is an observational, prospective study. Data were collected from 52 patients enrolled in a previous study [16]. All participants provided written informed consent prior to enrollment in the study.

Adult patients (≥18 years), presenting with primary and/or secondary occlusal disorders identified through digital occlusal analysis, were included in the study. Additional inclusion criteria included the presence of at least 10 functional occlusal units and the availability of a panoramic radiograph obtained within the previous six months.

Exclusion criteria comprised a history of occlusal adjustment or periodontal therapy within the past 12 months (excluding prophylactic visits), systemic conditions associated with periodontal disease (e.g., diabetes, hypertension, hematologic or renal disorders, and prior oncologic therapy), pregnancy, or inability/refusal to participate.

After analyzing previously reported prospective research on the topic of the change of the periodontal status [17,18,19,20,21,22,23,24,25,26], a sample size of at least 10 participants in each group that was followed up with was targeted. Participants were selected during routine dental consultations according to availability and eligibility.

Demographic data (age, gender), as well as clinical information regarding dental, prosthetic, periodontal, and occlusal status, were collected through structured interviews, clinical examination, and paraclinical assessment. Occlusal analysis was performed using a digital occlusal analyzer (OccluSense; Dr. Jean Bausch GmbH & Co. KG, Cologne, Germany), and orthopantomograms were evaluated for radiographic assessment. Occlusal analysis was performed as described in a previously published manuscript [16]. Occlusal trauma was assessed but not treated, which allowed observation of its influence without the bias of intervention.

Each patient underwent a full-mouth assessment using the Community Periodontal Index of Treatment Needs (CPITN), which evaluates periodontal status based on the presence of calculus and probing depth. The scoring system was as follows: score 0—healthy periodontium, score 1—bleeding on probing (BOP), score 2—presence of supra- or subgingival calculus, score 3—shallow periodontal pockets (PD ≤ 5.5 mm), and score 4—deep periodontal pockets (PD ≥ 6 mm) [27]. For this evaluation, the WHO periodontal probe (Hu-Friedy, Chicago, IL, USA) was used. For each patient, the CPITN index was reevaluated at 3 and 6 months.

Full examination was performed by a single calibrated examiner to ensure consistency and to avoid inter-examiner variability, i.e., X.A.N.

Based on the initial CPITN outcomes, all patients were indicated for initial (Phase I) periodontal therapy or no periodontal therapy, excluding any occlusal adjustments. As part of the treatment protocol, adjunctive use of chlorhexidine mouthwash (10 mL twice daily for 14 days) was recommended. The type of CHX mouthwash was chosen by the patients based on its availability at the drug store. Commercially available CHX products are typically found in local stores at concentrations of 0.05%, 0.1%, 0.12%, and 0.2%.

Data analysis was conducted using IBM SPSS Statistics, Version 22.0 (IBM Corp., Armonk, NY, USA). To compare groups, the Friedman and the Fisher’s exact tests were used. Statistical tests were selected according to data type and distribution. The statistical significance threshold was set at *p* < 0.05.

## 3. Results

The sample comprised 52 individuals, of whom 20 (38.5%) were male and 32 (61.5%) were female. Participants’ ages ranged from 23 to 72 years, with a mean age of 41.35 years.

**Figure 1:**
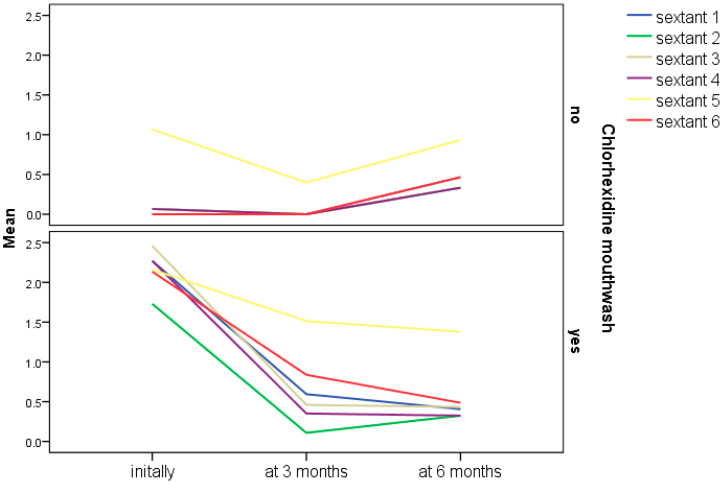
Evolution of CPITN index during the study for the participants who used, respectively, did not use Chlorhexidine mouthwash.

The variation in CPITN scores differed between individuals with and without an indication for CHX mouthwash (Figure 1). In the CHX group, a decrease in scores was observed during the first three months, followed by overall stabilization. In the non-CHX group, a slight reduction was recorded in sextant 5, while the remaining sextants showed unchanged scores; subsequently, between months 3 and 6, a slight upward trend was noted. Statistically significant changes in scores were observed in the CHX group, whereas no statistically significant variations were identified in the non-CHX group (Table 1).

At the three-month evaluation, a statistically significant improvement was observed in participants who used CHX mouthwash for all sextants, except for sextant 5 (Table 2), as well as for the overall score. Between the third and sixth months, the difference reached statistical significance only for sextant 6 and for the overall score (Table 2).

**Figure 2:**
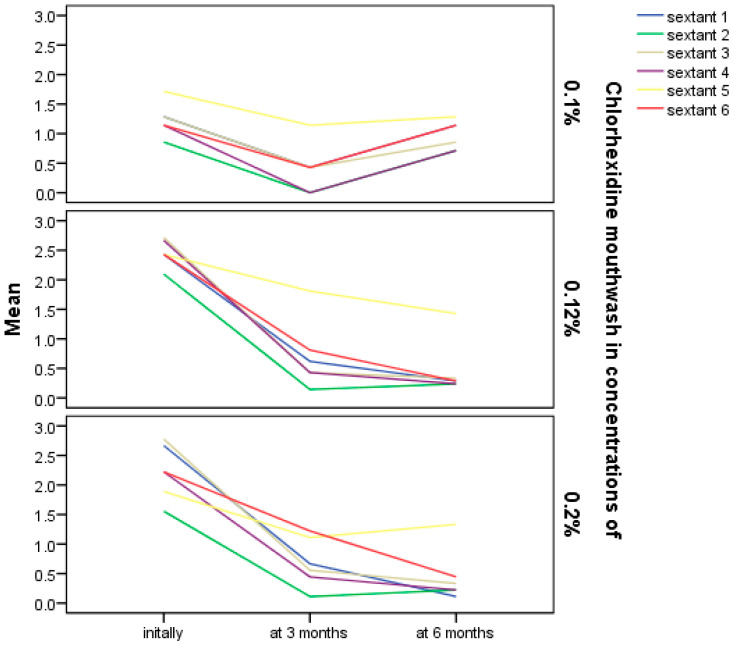
Evolution of CPITN index during the study for the participants who used CHX mouthwash in different concentrations.

Most participants (n = 21) used CHX mouthwash at a concentration of 0.12%, while fewer used 0.2% (n = 9) or 0.1% (n = 7). Comparative analysis of CPITN index variation (Figure 2), showed that use of CHX mouthwash at concentration of both 0.12% and 0.2% was associated with a greater decrease in CPITN index values, mainly after 3 months of use. Afterwards, values generally remained stable at the 6-month evaluation. In contrast, the use of CHX mouthwash at a concentration of 0.1% was associated with a trend of returning to the initial values at the 6-month evaluation. Statistically significant changes in CPITN index scores during the follow-up were observed for all sextants in the 0.12% CHX group, all except one sextant in 0.2% CHX group and none of the sextants in 0.1% CHX group (Table 3).

## 4. Discussion

The present study evaluated the impact of chlorhexidine (CHX) mouthwash on periodontal status among patients with occlusal trauma, without concurrent occlusal adjustment. Among available antiseptic mouthwashes, chlorhexidine mouthwash was selected due to its recognized gold-standard status in plaque control and its well-documented short-term benefits in reducing gingival inflammation [11,28]. Alternative formulations, such as povidone-iodine or herbal rinses, were excluded to preserve methodological consistency. The findings demonstrate statistically significant reductions in CPITN scores at the three-month mark in the CHX group across nearly all sextants (except sextant 5), with further significant improvement limited to sextant 6 and the overall score between months 3 and 6.

These outcomes align with existing evidence highlighting the role of CHX as a short-term adjunct to mechanical plaque control. For instance, a systematic review demonstrated that daily use of 0.2% CHX for 4–6 weeks reduces the clinical manifestations of gingivitis, reinforcing its benefit as a temporizing adjunct when mechanical hygiene is suboptimal [11]. Moreover, broader analyses show that CHX, when used alongside mechanical debridement, consistently reduces dental plaque and improves periodontal outcomes [28,29,30,31,32].

However, consistent with Cochrane and EFP guidelines, the clinical benefit of CHX is recognized as adjunctive and typically limited to short durations, since its effectiveness diminishes in moderate to severe periodontitis, particularly due to limited penetration into deep periodontal pockets [8,28,33].

Our observations indicate that improvements plateau become less pronounced after three months, which may reflect these inherent limitations. Moreover, broader literature cautions against prolonged use of CHX due to side effects including tooth staining, taste alteration, and mucosal irritation—primary factors compromising long-term compliance [13,15,21,34,35].

In the context of occlusal trauma, our findings suggest that improved biofilm control can yield substantial short-term periodontal gains, even when traumatic occlusal forces remain uncorrected. This supports the conceptual model that occlusal trauma alone does not initiate periodontitis but can exacerbate progression in the presence of inflammation. Our results imply that, by reducing microbial load via the use of CHX, the periodontal burden associated with occlusal overload may be partially mitigated.

Dental anxiety remains a significant barrier to care, delaying patient attendance and often resulting in more advanced periodontal disease at the time of presentation [36]. In this context, adjunctive use of chlorhexidine mouthwash may provide a temporary means of reducing microbial load and gingival inflammation in patients who postpone or avoid mechanical therapy due to fear or anxiety, thereby helping to prevent further periodontal deterioration.

Excessive occlusal load, combined with periodontal inflammation, can accelerate periodontal destruction. Preventive and curative treatment for trauma-related periodontitis involves addressing both the traumatic injury and the periodontal disease. Chlorhexidine (CHX) plays a significant role in the preventive effects of trauma associated with periodontitis, due to its antimicrobial properties, which reduce plaque accumulation and inhibit bacterial growth that may be exacerbated by occlusal trauma.

The decision to intervene should therefore be evidence-based and tailored to the individual patient’s functional and periodontal status.

Clinically, this underlines CHX’s utility as a bridging or adjunctive measure in patients where immediate occlusal correction is not feasible. Nonetheless, mechanical occlusal management remains essential for long-term periodontal stability, as evidenced in other reports within the field [11,37].

From a clinical perspective, the findings of this study support the use of CHX mouthwash as a short-term adjunctive therapy in patients with occlusal trauma and periodontal involvement. CHX can be particularly beneficial in situations where immediate occlusal adjustment is not feasible, for example, when patients require stepwise periodontal stabilization before complex prosthetic or orthodontic treatment. In such cases, prescribing CHX for 2–4 weeks may reduce inflammation and improve periodontal stability, thereby facilitating subsequent mechanical interventions. However, clinicians should be cautious regarding long-term use, given the risk of adverse effects and diminishing efficacy. Therefore, CHX should be regarded as a temporary adjunct, always combined with mechanical plaque control and followed, when indicated, by occlusal correction to ensure lasting periodontal health.

Our results align with those of Persson et al. [38], who used CPITN as a longitudinal index and reported early therapeutic gains that stabilized over time. Their longitudinal study of 3 years involved 123 patients with moderate to severe periodontitis [38]. The results after 1 year showed a greater reduction in CPITN index values in patients who underwent periodontal surgery in comparison with those who were treated only with non-surgical treatment, like our study, but at the 3-year follow-up, there was no difference between the groups [38]. The strong correlation between CPITN scores and anaerobic infection markers in the study by Muthukumar and Suresh [39] reinforces our methodology’s credibility. Additionally, findings by Tanik and Gül [27] suggest that full-mouth CPITN provides superior diagnostic validity compared to partial recordings, supporting the comprehensive sextant-based assessments we performed.

Our findings are consistent with broader evidence demonstrating that inflammation control is critical across patient populations, whether in children with systemic diseases or adults with functional risk factors such as occlusal trauma [40]. Previous studies have shown a strong association between dental and periodontal status, highlighting the multifactorial nature of periodontal disease [5,40,41]. In our study, improved biofilm control through chlorhexidine appears to partially counteract the inflammatory burden imposed by deflective occlusal contacts, underscoring the importance of an early and interdisciplinary approach.

### Limitations

The study’s observational design and limited sample size, particularly within subgroup analyses, may constrain the generalizability of the findings. The six-month follow-up period may be insufficient to capture long-term periodontal dynamics or the full impact of occlusal trauma. Future randomized controlled trials with larger cohorts, extended durations, and varied CHX formulations (e.g., sustained-release chips or gels) are warranted to validate and expand upon these results [42,43]. Moreover, future studies could also compare CHX with alternative antiseptic agents such as povidone-iodine or cetylpyridinium chloride to better delineate relative efficacy in patients with occlusal trauma.

## 5. Conclusions

This study provides evidence that CHX mouthwash significantly improves periodontal parameters in patients with occlusal trauma, particularly within the first three months of use. These findings reinforce the importance of CHX as a short-term adjunctive therapy for managing periodontal inflammation when immediate occlusal adjustment cannot be performed.

While CHX use resulted in clear short-term benefits, periodontal stability in the long term still requires correction of traumatic occlusal forces and consistent mechanical plaque control. CHX should therefore be regarded as a temporary, yet valuable component of a phased periodontal treatment plan, facilitating initial stabilization and creating favorable conditions for subsequent mechanical or surgical interventions. Therefore, while CPITN facilitated the detection of trends in our study, future investigations should complement it by including site-specific periodontal parameters to better capture disease dynamics.

## Figures and Tables

**Table 1:** *p*-value for CPITN index values during follow-up for the participants who used, respectively, did not use Chlorhexidine mouthwash.

*p*-Value for CPITN Values During Follow-Up	CHX (n = 37)	Without CHX (n = 15)
Sextant 1	<0.001	0.135
Sextant 2	<0.001	0.607
Sextant 3	<0.001	0.368
Sextant 4	<0.001	0.368
Sextant 5	0.007	0.206
Sextant 6	<0.001	0.135
Total score	<0.001	0.135
Friedman Test		

**Table 2:** Comparison of the efficiency of CHX for each sextant during follow-up, taking into consideration the CPITN values from baseline.

	CPITN						
		At 3 Months from Baseline			At 6 Months from Baseline		
		CHX n (%)	Without CHX n (%)	*p*	CHX n (%)	Without CHX n (%)	*p*
Sextant 1	improved	30 (81.1)	0 (0)	<0.001	8 (21.6)	0 (0)	0.089
	unchanged	7 (18.9)	15 (100)		27 (73)	13 (86.7)	
	worsened	0 (0)	0 (0)		2 (5.4)	2 (13.3)	
Sextant 2	improved	30 (81.1)	1 (6.7)	<0.001	2 (5.4)	0 (0)	>0.999
	unchanged	7 (18.9)	14 (93.3)		32 (86.5)	14 (93.3)	
	worsened	0 (0)	0 (0)		3 (8.1)	1 (6.7)	
Sextant 3	improved	31 (83.8)	1 (6.7)	<0.001	6 (16.2)	0 (0)	0.207
	unchanged	6 (16.2)	14 (93.3)		29 (78.4)	14 (93.3)	
	worsened	0 (0)	0 (0)		2 (5.4)	1 (6.7)	
Sextant 4	improved	31 (83.8)	1 (6.7)	<0.001	5 (13.5)	0 (0)	0.441
	unchanged	6 (16.2)	14 (93.3)		30 (81.1)	14 (93.3)	
	worsened	0 (0)	0 (0)		2 (5.4)	1 (6.7)	
Sextant 5	improved	16 (42.2)	6 (40)	0.758	9 (24.3)	2 (13.3)	0.336
	unchanged	16 (42.2)	8 (53.3)		24 (64.9)	9 (60)	
	worsened	5 (13.5)	1 (6.7)		4 (10.8)	4 (26.7)	
Sextant 6	improved	27 (73.0)	0 (0)	<0.001	10 (27)	0 (0)	0.029
	unchanged	10 (27.0)	15 (100)		25 (67.6)	13 (86.7)	
	worsened	0 (0)	0 (0)		2 (5.4)	2 (13.3)	
Total	improved	32 (86.5)	7 (46.7)	0.004	22 (59.5)	2 (13.3)	0.007
	unchanged	3 (8.1)	7 (46.7)		10 (27)	9 (60)	
	worsened	2 (5.4)	1 (6.7)		5 (13.5)	4 (26.7)	
Fisher‘s exact test							

**Table 3:** *p*-value for the CPITN index values for the participants who used CHX mouthwash in different concentrations.

*p*-Value for CPITN Values During Follow-Up	0.1% CHX	0.12% CHX	0.2% CHX
Sextant 1	0.074	<0.001	0.001
Sextant 2	0.074	<0.001	0.002
Sextant 3	0.099	<0.001	<0.001
Sextant 4	0.074	<0.001	<0.001
Sextant 5	0.538	0.025	0.196
Sextant 6	0.174	<0.001	0.001
Total score	0.062	<0.001	0.010
Friedman Test			

## Data Availability

The original contributions presented in this study are included in this article. Further inquiries can be directed to the corresponding authors.
